# BLCA prognostic model creation and validation based on immune gene-metabolic gene combination

**DOI:** 10.1007/s12672-023-00853-6

**Published:** 2023-12-16

**Authors:** Shao-Yu Yue, Di Niu, Xian-Hong Liu, Wei-Yi Li, Ke Ding, Hong-Ye Fang, Xin-Dong Wu, Chun Li, Yu Guan, He-Xi Du

**Affiliations:** 1 Department of Urology, the First Affiliated Hospital of Anhui Medical University, Anhui Medical University, No. 218 Jixi Road, Hefei, 230022 Anhui People’s Republic of China; 2 Institute of Urology, Anhui Medical University, Hefei, Anhui People’s Republic of China; 3 Anhui Province Key Laboratory of Genitourinary Diseases, Anhui Medical University, Hefei, Anhui People’s Republic of China

**Keywords:** Bladder cancer, Prognostic model, Immunogene, Integrated biomarker approach, Prognosis

## Abstract

**Background:**

Bladder cancer (BLCA) is a prevalent urinary system malignancy. Understanding the interplay of immunological and metabolic genes in BLCA is crucial for prognosis and treatment.

**Methods:**

Immune/metabolism genes were extracted, their expression profiles analyzed. NMF clustering found prognostic genes. Immunocyte infiltration and tumor microenvironment were examined. Risk prognostic signature using Cox/LASSO methods was developed. Immunological Microenvironment and functional enrichment analysis explored. Immunotherapy response and somatic mutations evaluated. RT-qPCR validated gene expression.

**Results:**

We investigated these genes in 614 BLCA samples, identifying relevant prognostic genes. We developed a predictive feature and signature comprising 7 genes (POLE2, AHNAK, SHMT2, NR2F1, TFRC, OAS1, CHKB). This immune and metabolism-related gene (IMRG) signature showed superior predictive performance across multiple datasets and was independent of clinical indicators. Immunotherapy response and immune cell infiltration correlated with the risk score. Functional enrichment analysis revealed distinct biological pathways between low- and high-risk groups. The signature demonstrated higher prediction accuracy than other signatures. qRT-PCR confirmed differential gene expression and immunotherapy response.

**Conclusions:**

The model in our work is a novel assessment tool to measure immunotherapy’s effectiveness and anticipate BLCA patients’ prognosis, offering new avenues for immunological biomarkers and targeted treatments.

**Supplementary Information:**

The online version contains supplementary material available at 10.1007/s12672-023-00853-6.

## Introduction

Bladder cancer (BLCA), commonly known as urothelial carcinoma, is a prevalent urinary system malignancy. In 2022, China recorded 91,893 newly diagnosed cases of BLCA, with 71,002 cases reported in males and 20,891 cases in females [1]. According to estimates, the incidence of BLCA in men will continue to be higher than in women by 2030 [2]. Furthermore, cigarette smoking, occupational exposure, and exposure to carcinogens are recognized as significant contributors to the pathogeny and progression of BLCA [3, 4]. Currently, radical cystectomy (RC) in conjunction with lymph node dissection is considered the established therapeutic approach for individuals diagnosed with high-risk muscle-invasive bladder cancer (MIBC) and non-muscle invasive bladder cancer (NMIBC) in the absence of distant metastasis[5]. Chemotherapy using cisplatin is still the most common and effective treatment option for people with metastatic BLCA [6]. Nevertheless, there remains a dearth of widely accepted prognostic tissue biomarkers for BLCA [6]. Novel paths and strategies are emerging to improve predictive assessment and therapeutic interventions in the management of this disease as the field of genetic research in BLCA progresses [7–9]. Therefore, to increase the effectiveness of BLCA treatment, it is extremely important to uncover early diagnostic and prognostic biomarkers by examining the GEO and TCGA databases.

The relationship between immune genes and BLCA is intricate and multifaceted. Immune gene dysregulation can help in the emergence of BLCA [10–13]. Numerous studies have discovered alterations in immune gene expression and immunocyte infiltration in BLCA [14–18]. These modifications may affect the production of chemokines and cytokines, immune surveillance, immune cell infiltration into tumors, and other elements of the immunological response [19]. In BLCA, several immune genes have been thoroughly investigated, including those that encode immune checkpoint molecules, including PD-L1, PD-1, and CTLA-4 [14, 20–23]. These genes can become dysregulated, allowing tumor cells to avoid immune monitoring and proliferate. Additionally, immunocytes, such as myeloid, natural killer (NK), T, and B cells, infiltrate the tumor microenvironment in BLCA [24–26]. Interactions between these immunocytes and tumor cells could influence tumor growth, invasion, and response to treatment. Immunotherapy, which includes adoptive cell therapies and immunological checkpoint inhibitors, has emerged as a promising approach for treating BLCA. These treatments target immune genes and modify the immune response to improve anti-tumor immune activity and patient outcomes. Understanding this association is critical for establishing successful immunotherapeutic treatments and improving BLCA prognosis.

The correlation between metabolic genes and BLCA is intricate and interrelated, with aberrant expression or mutations in metabolic genes potentially contributing to the etiology and progression of BLCA. According to studies, changing the expression of metabolic genes can affect energy and substance metabolism, supplying the nutrients and biochemical components required for long-term tumor cell growth [27–29]. Additionally, dysregulated expression of metabolic genes may induce oxidative stress, DNA damage, and cellular apoptosis, thereby promoting BLCA development [30–34].

Numerous metabolic genes have been thoroughly studied in the context of BLCA. For example, genes involved in glucose metabolism, such as phosphatase and tensin homolog (PTEN) [35, 36] and glucose transporter 1 (GLUT1) [37–39], are implicated in the onset and progression of BLCA. Additionally, certain metabolic regulators and enzymes, such as lactate dehydrogenases A (LDHA) and B (LDHB), are essential in controlling the metabolism and the tumor cell energy pathways [40–42]. Understanding the complex interaction between metabolic genes and BLCA is critical for understanding the underlying mechanisms that drive disease development and progression. Such knowledge lays the foundation for the development of specialized therapeutic approaches. Further investigation will clarify the relationship between metabolic genes and BLCA, presenting new opportunities for individualized treatment plans and prognosis evaluation.

This study systematically analyzed the expression patterns and potential functions of immunological genes and metabolic genes in bladder cancer (BLCA) by employing a comprehensive integration of bioinformatics methods. A prognostic feature was developed and validated based on seven relevant genes, exhibiting a high accuracy in the prognosis prediction of BLCA patients. The predictive feature was also identified as an independent prognostic indicator, showing a correlation with immune cell infiltration. These findings suggest that an effective BLCA treatment may involve combining immunological and metabolic genes.

## Materials and methods

### Data acquisition and processing

We examined 614 samples from the TCGA-BLCA dataset, obtained from The Cancer Genome Atlas (TCGA) website (https://portal.gdc.cancer.gov/), containing 72 normal and 542 tumor samples. Among these tumor samples, 398 patients with overall survival information were assigned randomly in a 7:3 ratio to the TCGA training set (279 samples) and the TCGA testing set (119 samples). This division was carried out in preparation for future label development and confirmation. Data on mutations and clinical features were obtained from the TCGA database. For extracting mutation information for each sample, the mutation data were analyzed using the Strawberry Perl software (https://strawberrysw.com/). For each patient, the tumor mutation burden (TMB) score was determined by dividing the total number of mutations by the total number of covered bases and multiplying the result by 10^6^.

In addition, we extracted the GSE40914 dataset from the GEO (Gene Expression Omnibus) database (https://www.ncbi.nlm.nih.gov/geo/) and added it to the TCGA dataset. After normalizing, the integrated dataset was used to validate the obtained signatures externally. To supplement our analysis, we obtained 2483 immune-related genes from the ImmPort database (https://www.immport.org/shared/home) and 948 metabolism-related genes from the GSEA metabolic pathways. Further, the expression profiles were extracted from the TCGA-BLCA expression dataset.

### Differential analysis and NMF clustering analysis

Differential analysis was conducted on 2,652 immune and metabolism-related genes (IMRG). The analysis employed criteria such as |logFC|> 1, P < 0.05, and FDR < 0.05 as thresholds to identify genes exhibiting differential expression. Genes differentially expressed and shared between the TCGA and GEO datasets were extracted to improve the study by integrating data from the GEO dataset. The shared set of differentially expressed genes was then subjected to univariate Cox regression analysis to find genes correlated with prognosis (P < 0.05). The "NMF" tool in the R programming language performed NMF (non-negative matrix factorization) analysis on the discovered prognostic-associated genes. The “survival” package in R was utilized to conduct the survival analysis, and Kaplan–Meier curves were produced to show each subgroup’s overall survival (OS). The Log-rank test evaluated the variations in survival results between the groupings.

### Prognostic factor analysis and characterization of the immune Microenvironment across distinct subgroups

The tumor microenvironment (TME) scores for individual samples in the TCGA-BLCA cohort were determined using the R-based ESTIMATE program. We evaluated the different immune and stromal cell infiltration across several subgroups using the “MCPcounter” package in R. Additionally, comparative studies across the subgroups have been conducted to assess variations in immune, estimation, and stromal scores.

### Development of risk prognostic signature

Further, a univariate Cox regression analysis was conducted on the immunological and metabolism-related genes (IMRGs) that were common between the GEO and TCGA datasets in the TCGA training cohort. Prognostic-associated IMRGs were identified based on their statistical significance in the analysis. To mitigate the risk of overfitting, we employed the LASSO regression method to refine further and select the most relevant IMRGs among the prognostic-associated genes. The best gene combination to produce a risk prognostic signature was then determined using multivariate Cox regression (MCR) analysis, limiting the possibility of confounding effects. We acquired the risk score formula (as given below) for the prognostic signature based on the findings of the MCR study.

$$ \begin{aligned}   {\text{Riskscore}} =  &\, {\text{expression }}_{{{\text{POLE}}2}}  \times \beta _{{{\text{POLE}}2}}  + {\text{expression }}_{{{\text{AHNAK}}}}  \times \upbeta _{{{\text{AHNAK}}}}  \\     &  + \,{\text{expression }}_{{{\text{SHMT}}2}}  \times \upbeta _{{{\text{SHMT}}2}}  + {\text{expression }}_{{{\text{NR}}2{\text{F}}1}}  \times \upbeta _{{{\text{NR}}2{\text{F}}1}}  \\     &  + \,{\text{expression }}_{{{\text{TFRC}}}}  \times \upbeta _{{{\text{TFRC}}}}  + {\text{expression }}_{{{\text{OAS}}1}}  \times \upbeta _{{{\text{OAS}}1}}  \\     &  + \,{\text{expression }}_{{{\text{CHKB}}}}  \times \upbeta _{{{\text{CHKB}}}}  \\  \end{aligned}  $$

### Independence factor analysis

UniCox and multiCox regression analyses examined independent covariates, such as risk scores and clinical traits. To determine each factor’s level of predictability, ROC analysis was employed.

### Nomogram construction and calibration plot

Kaplan–Meier (K–M) curves were created to examine OS to assess this feature's prognostic importance. In order to evaluate the predictive efficacy of this feature, time-dependent ROC (receiver operating characteristic) curves were developed for survival at 1-year, 3-year, and 5-year intervals. To evaluate the predictive effectiveness of this feature, the area under the curve (AUC) values were generated.

### Functional enrichment analysis

Investigating the Underlying Biological Mechanisms between low- and high-risk groups: The “c2.cp.kegg.symbols.gmt” file retrieved from the MSigDB database was used to perform GSEA (Gene Set Enrichment Analysis). The “GSVA” package in R software was utilized to perform the enrichment analysis of gene sets and find the enriched pathways for each group.

### Investigating the immunological microenvironment

The “MCPcounter” program was used to calculate the relationship between risk score and 10 immune cell infiltrations, and 10 immune cell infiltrations were compared between both groups. In addition, correlations between risk scores and expression levels of 12 common immune checkpoint genes were calculated. In the meantime, differential analysis of the 12 immunological checkpoint genes between both groups was performed using the Limma software.

### Analysis of immunotherapy response and assessment of somatic mutations

We displayed the association circles between 10 immunocytes, risk score, microsatellite instability (MSI), tumor mutational burden (TMB), and these variables in LUAD. We plotted K-M curves to explore the correlation between TMB level and prognosis. We obtained the immunophenotype scores (IPS) of LUAD patients from The Cancer Immunome Atlas (TCIA) (https://tcia.at/) to examine the role represented by the IMRG signature in the immunotherapy response. After that, the IPS of the low- and high-risk groups were analyzed.

### Comparison with other studied signatures

The C index of the IMRG signature was compared with that of other signatures to assess how well it predicted outcomes compared to different signatures. The ROC and K-M curves of the IMRG signature and other signatures were also compared.

### Real-time quantitative polymerase chain reaction (RT-qPCR)

Following the manufacturer's instructions, total RNA was isolated from human bladder tissues using the TRIzol reagent (Invitrogen, USA). The PrimeScriptTM RT kit and gDNA Eraser (RR047A, Takara) were used for cDNA first-strand synthesis, and the TB Green Premix Ex Taq II kit (RR82WR, Takara) was employed for reverse transcription analysis. An ABI 7500 PCR equipment (Applied Biosystems, USA) was used for each qPCR. The human GAPDH enzyme was utilized as an internal control, with a volume of 20 μL per tube. Data analysis involved the application of the 2^−ΔΔCt^ formula to each sample. The primer sequences are provided in Supplement Table 1.

### Western blotting

The levels of protein expression of AHNAK, POLE2, SHMT2, NR2F1, TFRC, OAS1 and CHKB were measured by western blotting. AHNAK, POLE2, SHMT2, NR2F1, TFRC, OAS1 and CHKB were separated by employing sds–polyacrylamide gels (10%), and were transferred onto NC membranes (Bio-Rad, Hercules, USA). After one-hour blocking in 5% BSA at indoor temperature, membranes were subjected to the subsequent incubation of following antibodies at 4 °C overnight: AHNAK antibody (1:500, DF10323, Affinty), TFRC antibody (1:500, AF5343, Affinty), SHMT2 antibody (1:500, DF6347, Affinty), OAS1 antibody (1:500, DF7760, Affinty), CHKB antibody (1:500, DF3477, Affinty), POLE2 antibody (1:500, DF9446, Affinty) and NR2F1 antibody (1:500, DF15501, Affinty). GAPDH antibody (1:5000, AF7021, Affinty). After three TBST washes, membranes were treated with secondary IgG antibodies (anti-rabbit or anti-mouse, 1:3000, Elabscience) for 1 h. Final, enhanced chemiluminescence (ECL) reagents (Advansta, K-12043, USA) and a film (ChemiScope 5600, Hengmei Technology, China) were used to display the protein bands.

## Results

### Identification of DEGs and two molecular subtypes

**Fig. 1:**
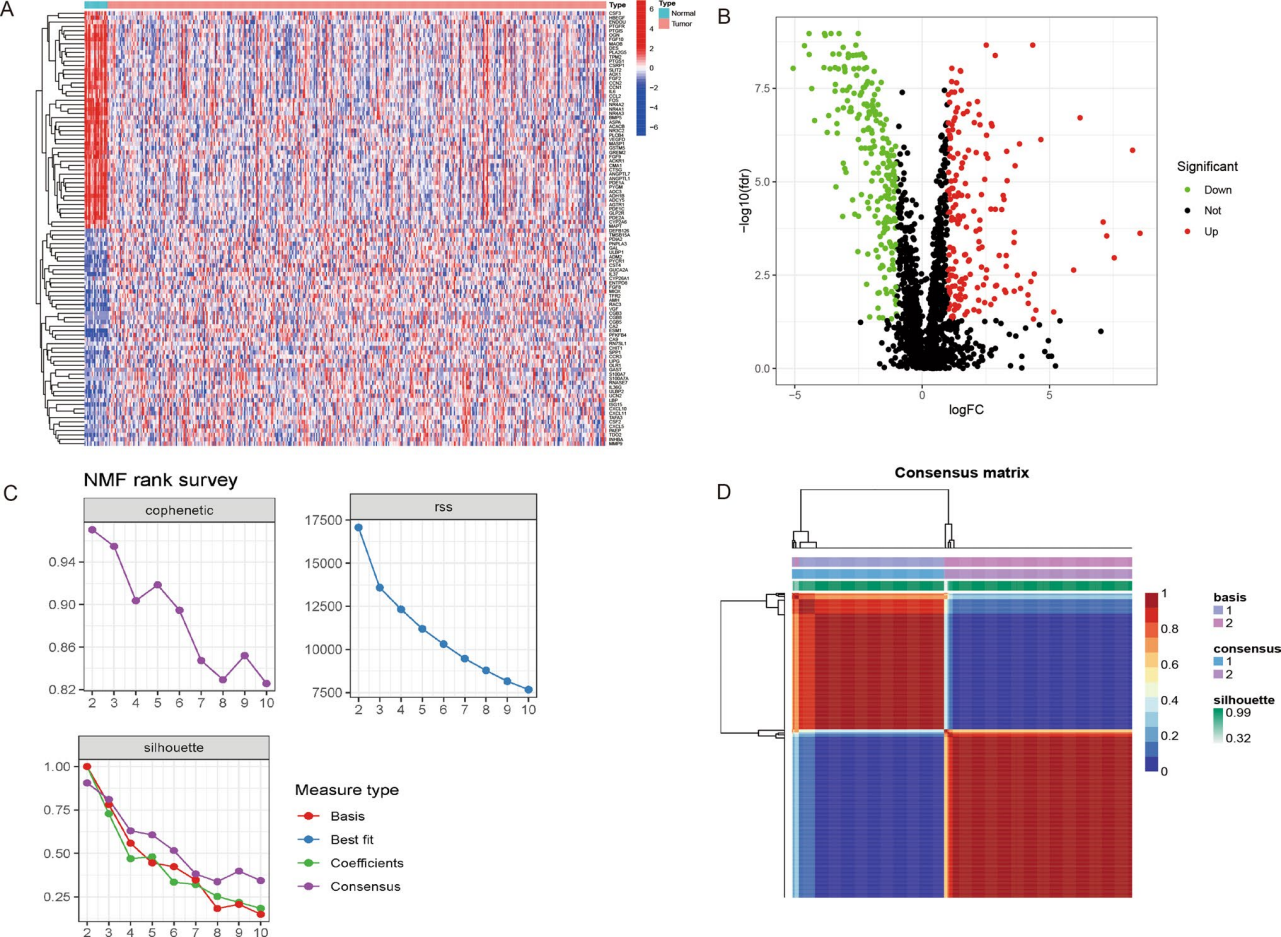
Identification of DEGs and two molecular subtypes. **A** A heatmap visualizing the differential expression patterns of immune and metabolism-related genes (IMRGs) in tumor and normal tissues. **B** A volcano graphic illustrating the importance of IMRGs’ differential expression in tumor and healthy tissues. **C** The workflow illustrates the sequential steps involved in NMF clustering analysis. **D** A heatmap shows the non-negative matrix factorization (NMF) clustering analysis results

The differential analysis yielded 986 immune and metabolism-related genes (IMRGs) with differential expression, comprising 252 downregulated genes and 734 upregulated genes. Within this set, 383 genes were identified as common in both the GEO and TCGA datasets (Fig. 1A, B). 49 IMRGs related to prognosis were found when these 383 genes underwent univariate Cox regression analysis. The TCGA-LUAD cohort was subjected to clustering using the NMF program using the expression profiles of these 49 IMRGs associated with prognosis. The clustering effects were evaluated for k values ranging from 1 to 10. Notably, when k = 2, there was a significant decrease in the consensus correlation coefficient, and the heatmap displayed more uniform color patterns across the groups (Fig. 1C, D). As a result, the LUAD samples were classified into three distinct subgroups: C1 (n = 172) and C2 (n = 226).

### Analysis of survival and immune infiltration

**Fig. 2:**
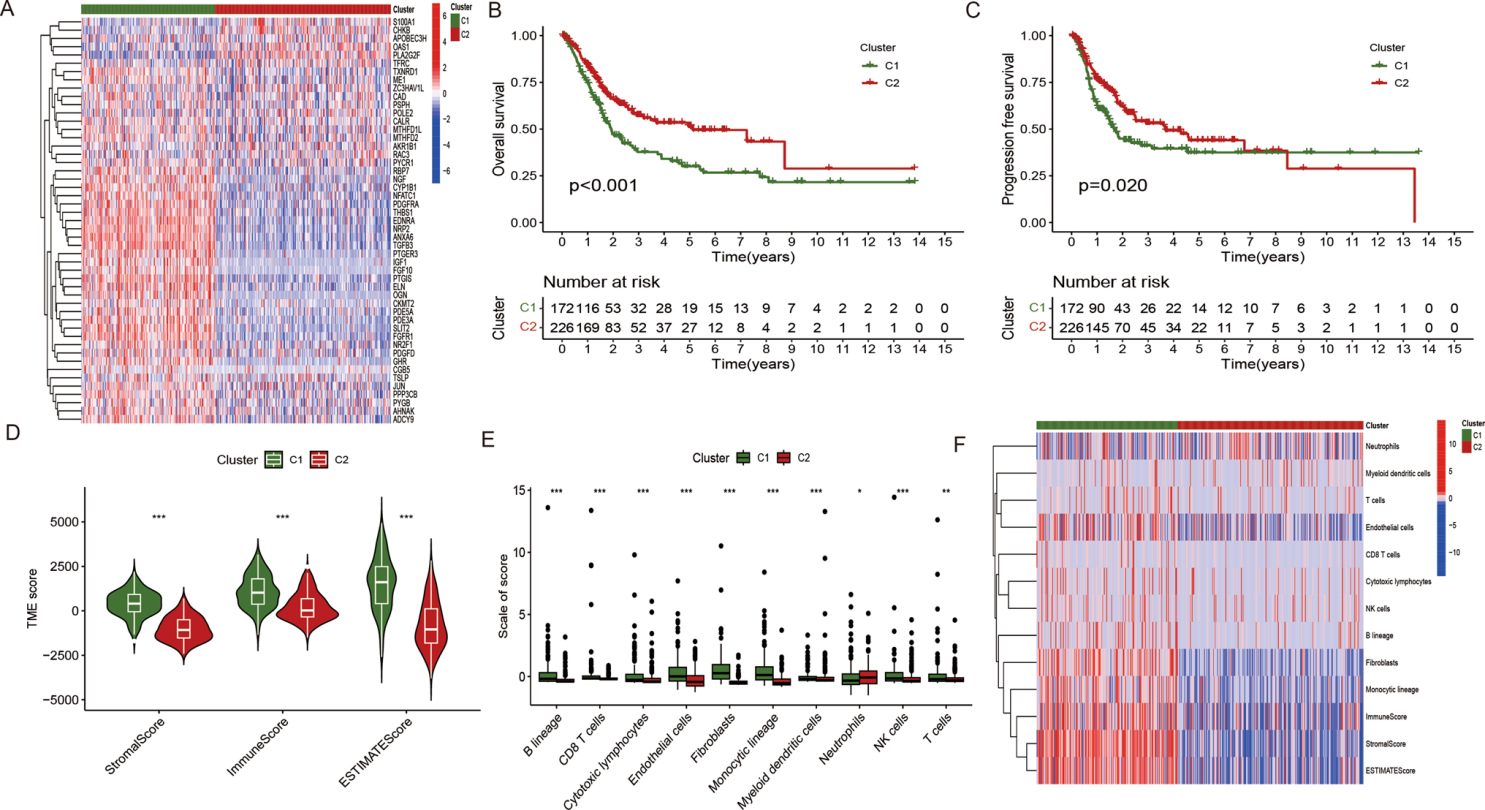
Analysis of survival and immune infiltration. **A** Heat map illustrating the expression patterns of 49 IMRGs associated with prognosis across different subgroups. **B** KM curve depicting the overall survival (OS) of the subgroups. **C** Kaplan–Meier curve illustrating the progression-free survival (PFS) of the subgroups. **D** Levels of infiltration by 10 immune cell types in each subgroup. **E** Immune score, estimate score, and stromal score for each subgroup. **F** Heat map displaying the infiltration levels of 10 immunocyte species in each subgroup

Significant differential gene expression was observed in 49 genes between the two subgroups (Fig. 2A). According to the K-M survival analysis (P < 0.001), the two subgroups had a significant difference in overall survival. While subgroup C1 showed the worst outcome, subgroup C2 exhibited a positive prognosis (Fig. 2B). Additionally, a significant difference in progression-free survival (PFS) between the two categories was discovered (P = 0.02) (Fig. 2C). Subgroup C1 revealed higher tumor microenvironment (TME) values, stromal scores, ESTIMATE scores, and immunological scores (Fig. 2D). T cells, NK cells, Myeloid dendritic cells, Monocytic lineage, Fibroblasts, Endothelial cells, Cytotoxic lymphocytes, CD8 T cells, and B lineage exhibited higher infiltration scores in subgroup C1 (P < 0.05), indicating a more active anti-tumor immune cell response in C1 subgroup. Most immune cell infiltration scores were lower in subgroup C1(Fig. 2E, F).

### Development and validation of risk prognostic signatures in BLCA

**Fig. 3:**
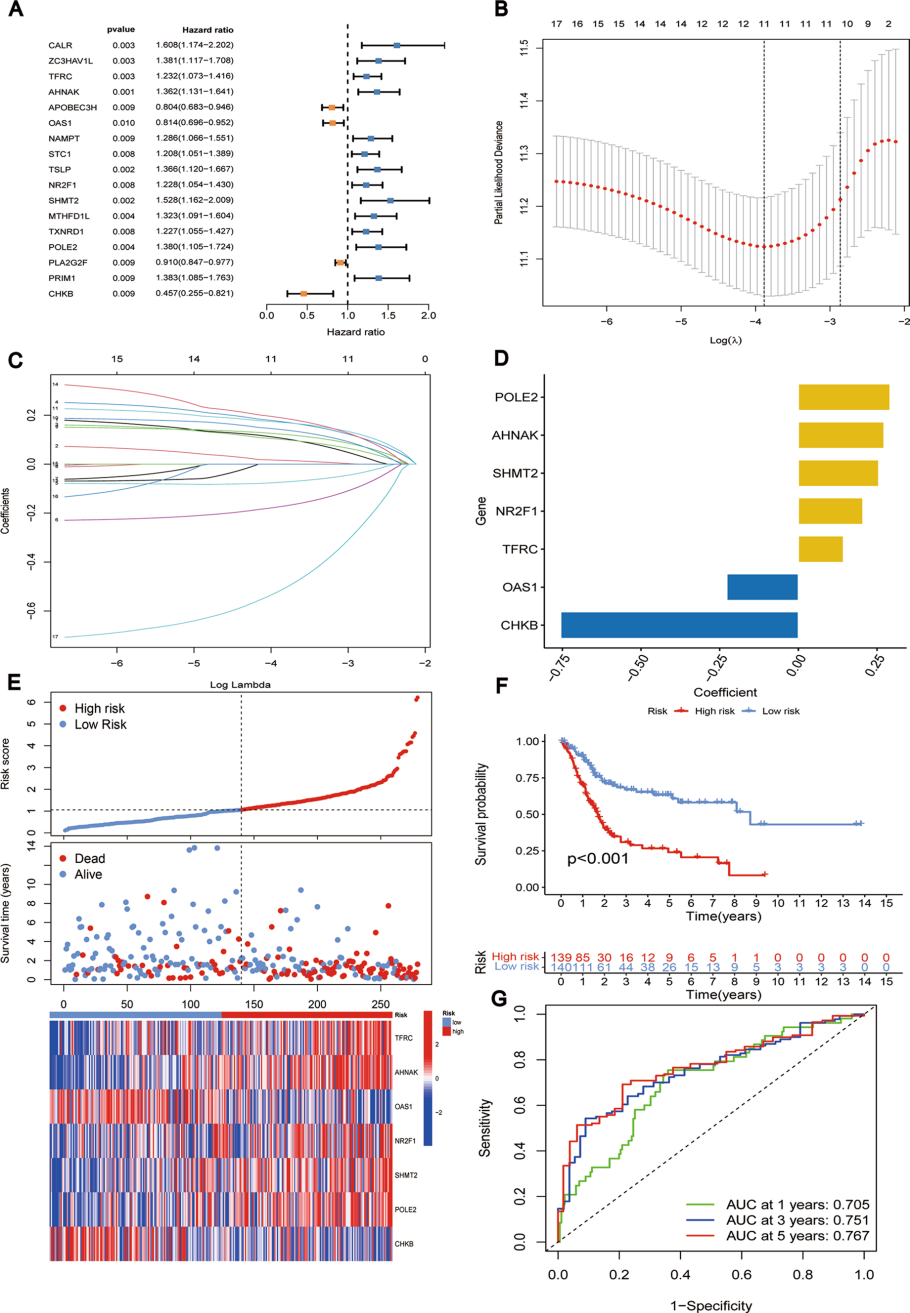
Development and confirmation of risk prognostic signatures in BLCA. **A** A univariate Cox regression analysis of 383 IMRGs revealed 17 predictive genes. **B**, **C** Eleven IMRGs were selected by LASSO regression. **D** MCRcoefficients of 7 genes in the signature. **E** Risk score curves, risk state dot plots, and 7 prognostic gene expression heat maps at TCGA training cohort. **F** KM curve of OS in TCGA training cohort. **G** AUC values of 1-year, 3-year, and 5-year ROC curves were calculated using TCGA data in the training cohort

A univariate Cox regression study of 383 IMRGs in the TCGA training cohort revealed 17 predictive genes (P < 0.01) (Fig. 3A). The 30 IMRGs were subjected to LASSO regression analysis to reduce overfitting, and 11 IMRGs were picked as a consequence (Fig. 3B). The 11 IMRGs were then subjected to MCRanalysis, which finally resulted in identifying a risk prognostic signature consisting of 7 genes. On the ground of the coefficients of these 7 genes (Fig. 3C, D), the risk score formula of the signature was as follows:

$$ \begin{aligned}   {\text{Riskscore}}{\mkern 1mu} {\mkern 1mu} \, =  & {\mkern 1mu} {\mkern 1mu} {\text{expression}}_{{{\text{POLE2}}}} *\upbeta _{{{\text{POLE2}}}}  + {\mkern 1mu} {\text{expression}}_{{{\text{AHNAK}}}} *\upbeta _{{{\text{AHNAK}}}} {\mkern 1mu}  \\     &  + \,{\mkern 1mu} {\mkern 1mu} {\text{expression}}_{{{\text{SHMT2}}}} *\upbeta _{{{\text{SHMT2}}}} {\mkern 1mu}  + \,{\mkern 1mu} {\text{expression}}_{{{\text{NR2F1}}}} *\upbeta _{{{\text{NR2F1}}}} {\mkern 1mu}  \\     &  + {\mkern 1mu} {\mkern 1mu} \,{\text{expression}}_{{{\text{TFRC}}}} *\upbeta _{{{\text{TFRC}}}} {\mkern 1mu}  + {\mkern 1mu} \,{\text{expression}}_{{{\text{OAS1}}}} *\upbeta _{{{\text{OAS1}}}} {\mkern 1mu}  \\     &  + \,{\mkern 1mu} {\text{expression}}_{{{\text{CHKB}}}} *\upbeta _{{{\text{CHKB}}}}  \\  \end{aligned}  $$

**Fig. 4:**
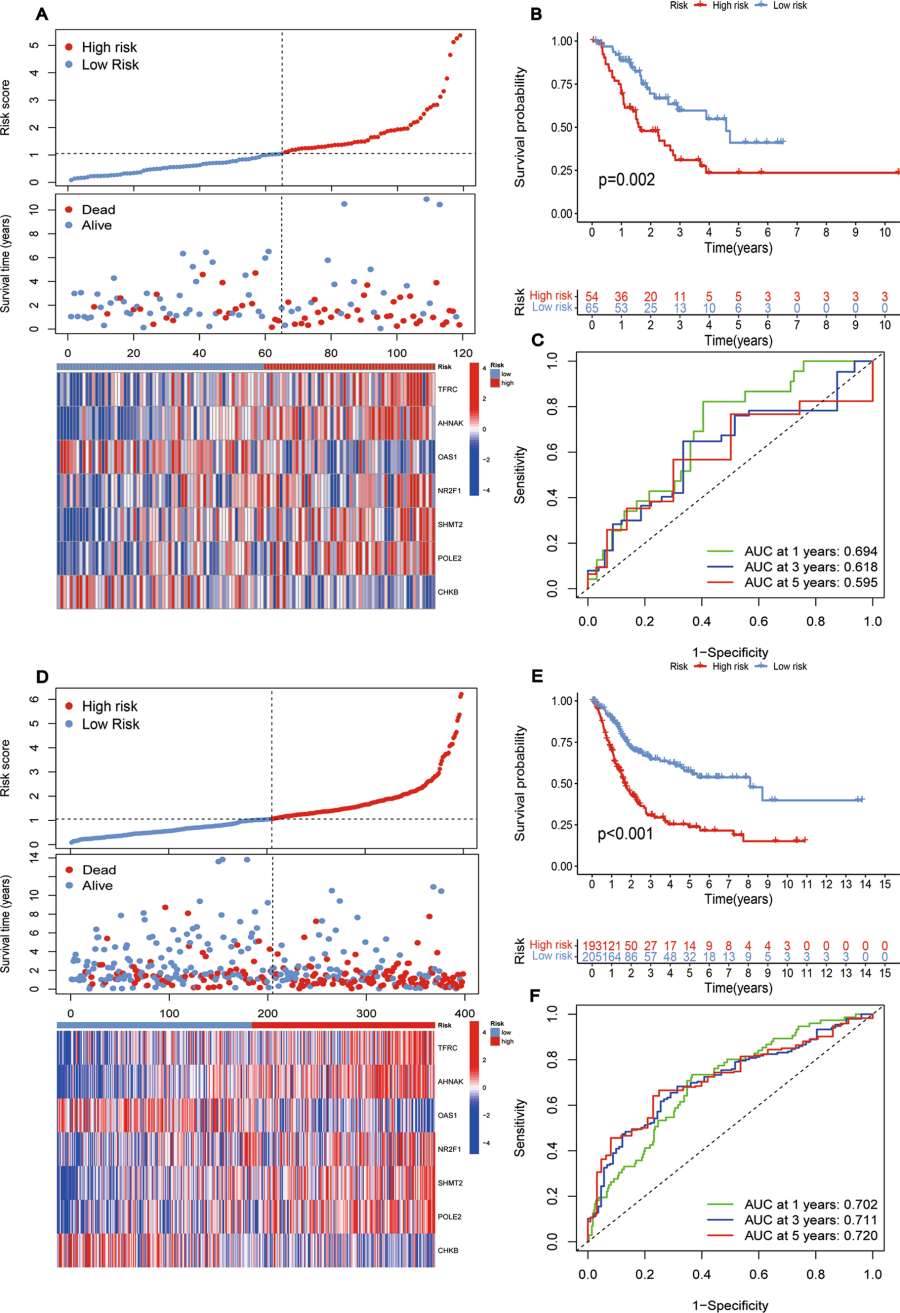
**A** Risk score curves, risk state dot plots, and 7 prognostic gene expression heat maps at TCGA test cohort. **B** KM curve of OS in TCGA testing cohort. **C** AUC values of 1-year, 3-year, and 5-year ROC curves were calculated using TCGA data in the testing cohort. **D** Risk score curves, risk state dot plots, and 7 prognostic gene expression heat maps at TCGA all cohorts. **E** KM curve of OS in TCGA all cohort. **F** AUC values of 1-year, 3-year, and 5-year ROC curves were calculated using TCGA data in all cohorts

**Fig. 5:**
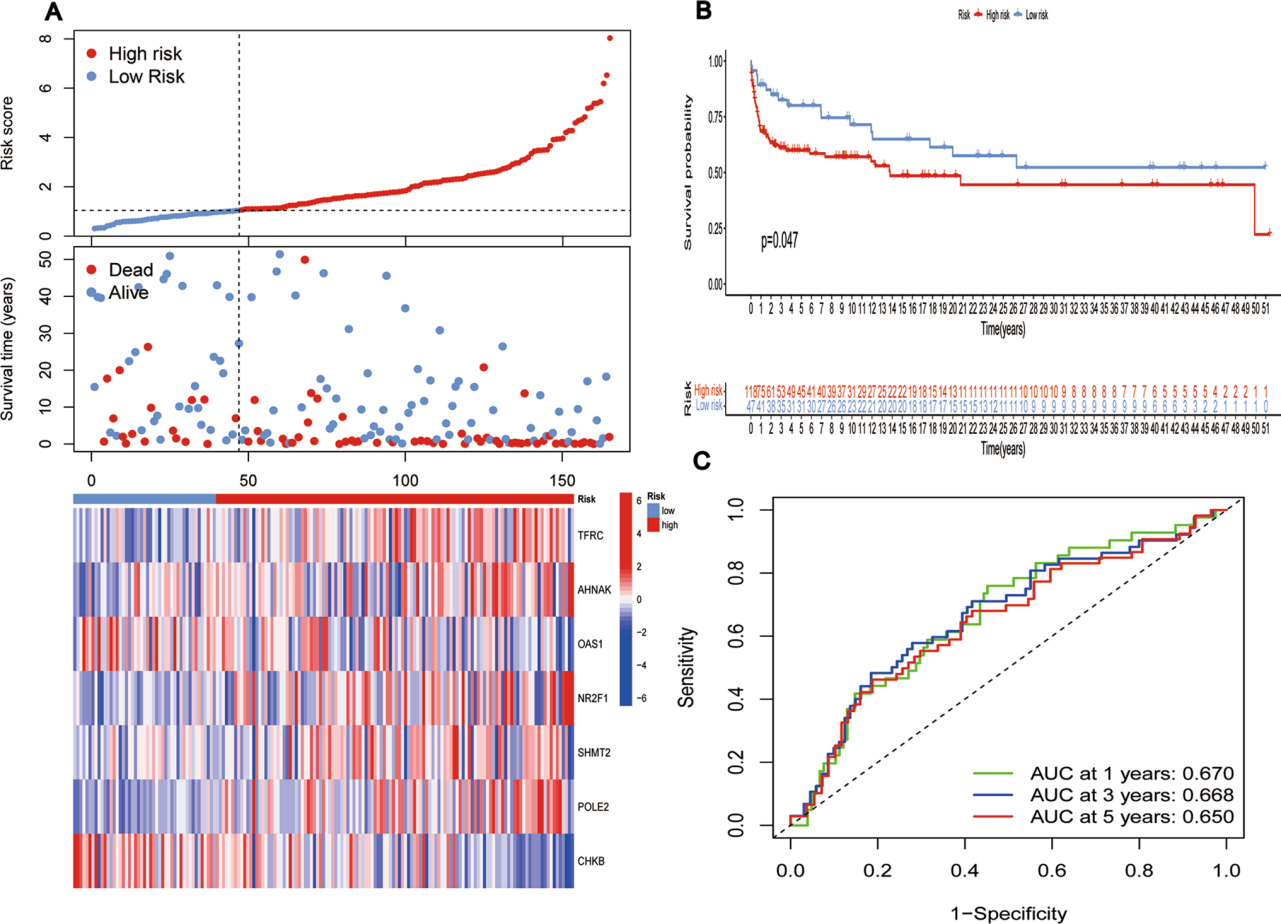
**A** Risk score curves, risk state dot plots, and 7 prognostic gene expression heat maps at the GEO cohort. **B** KM curve of OS in GEO cohort. **C** AUC values of 1-year, 3-year, and 5-year ROC curves were calculated using GEO in the testing cohort

As mentioned above, the formula can be used to calculate the risk scores for each patient. Patients were categorized into low- and high-risk groups based on the median score. The prognosis of BLCA patients declined as the risk score rose, as evidenced by risk score curves and survival status plots (Fig. 3E**, **Figs. 4A, D and 5A). OAS1 and CHKB expression levels negatively correlated with risk score, pointing to their preventive functions. Conversely, the expression levels of POLE2, AHNAK, SHMT2, NR2F1, and TFRC were positively related to the risk score, indicating their roles as risk factors. The predictive effectiveness of this signature was shown by a survival prognosis analysis that revealed a strong association (P < 0.001) between the high-risk group and poor outcome.

Furthermore, consistent results were obtained in the TCGA training cohort (P < 0.001, Fig. 3F), TCGA testing cohort (P = 0.002, Fig. 4B), the entire TCGA cohort (P < 0.001, Fig. 4E), and the GEO cohort (P = 0.047, Fig. 5B). The overall TCGA cohort, TCGA testing cohort, TCGA training cohort (0.720, 0.711, 0.702), and the GEO cohort (0.670, 0.668, 0.650) all revealed higher AUC values for the 1-, 3-, and 5-years ROC curves (Fig. 3G**, **Figs. 4C, F, and 5C). These findings show this the risk prognostic signature possesses stronger predictive performance.

### Confirmation of risk prognostic signatures as independent prognostic factors

**Fig. 6:**
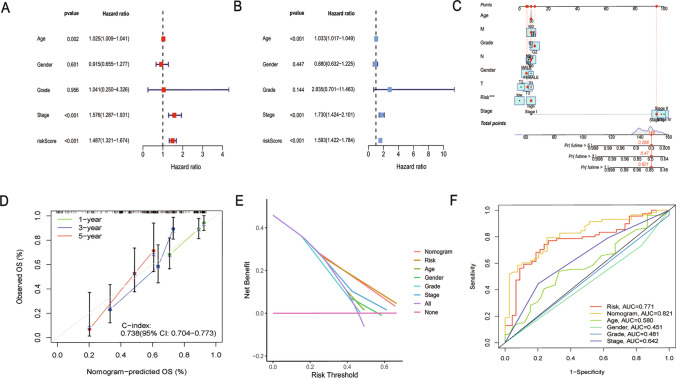
Validation of risk prognostic signatures as independent prognostic factors. **A** Univariate Cox regression. **B** Multivariate Cox regression. **C** Nomogram constructed. **D** Calibration curve for nomogram. **E** DCA curve. **F** ROC curve

Association of Risk Score with Bladder Cancer Development: Analysis of multifactorial Cox regression with a risk score of < 0.001 and HR of 1.487 (1.321–1.674) furthermore to a univariate Cox regression analysis with a risk score of < 0.001 and an HR of 1.593 (1.422–1.784) established the independent impact of risk score on BLCA patients' prognosis (Fig. 6A, B). The predicted accuracy was greatly improved by combining the risk score with clinicopathological characteristics (gender, age, N, T, M, and stage), bringing it closer to actual results (Fig. 6C, D). Moreover, decision curve analysis (DCA) demonstrated the superior concordance of the nomogram and risk score with clinical decision-making (Fig. 6E). The nomogram demonstrated the highest predictive ability, followed by the risk score, comparing the 3-year ROC values for the nomogram, risk score, and clinicopathological variables (stage, age, sex) (Fig. 6F).

### Relationship between risk prognostic models and clinically relevant features

**Fig. 7:**
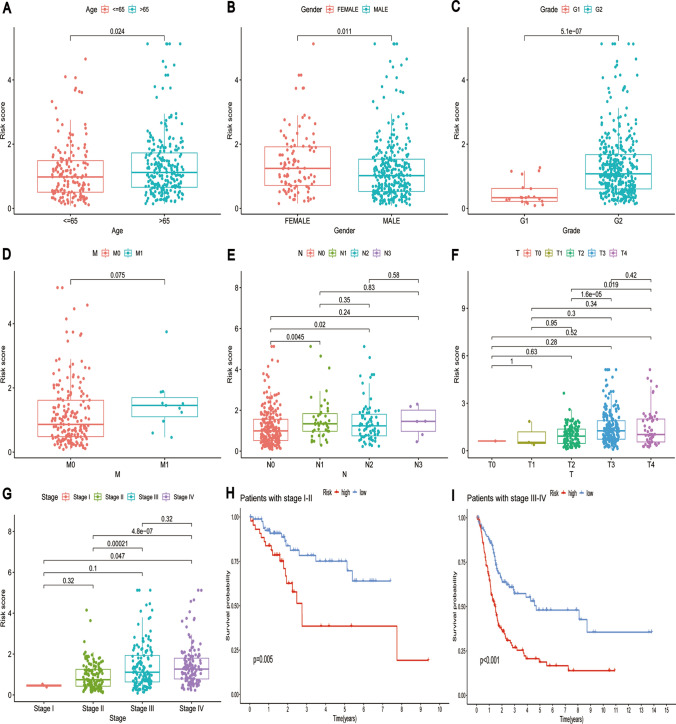
Relationship between risk prognostic models and clinically relevant features. **A**–**G** The correlation between the risk score and clinicopathological characteristics, including age, gender, T, N, M, and overall stage, was assessed. **H**–**I** Survival prognostic curves in Stage I–II Stage III–IV

Patients with bladder cancer 65 years of age and older experienced higher risk scores, which may indicate a poor prognosis (Fig. 7A). Additionally, women are more susceptible to bladder cancer, suggesting that gender may play a role in its development (Fig. 7B). Among the grade classifications in bladder cancer, G2 patients exhibit higher risk scores, implying a greater propensity for disease progression and adverse prognostic outcomes (Fig. 7C). Furthermore, the provided data emphasizes variations in the M stage (distant metastasis) of bladder cancer showed no significance across different pathological stages. In the N stage (lymph node metastasis), differences are observed between N0 and N1 and N0 and N2.

Similarly, variations exist between T2 and T3 and T2 and T4 in the T stage (primary tumor spread) (Fig. 7D–F). Moreover, the advanced-stage (III-IV) and early-stage (I–II) bladder cancers carry prognostic significance (Figs. 7G–I). This suggests that patients' prognoses may fluctuate noticeably depending on the stage of their bladder cancer. These outcomes have significant clinical implications for choosing therapeutic modalities and caring for bladder cancer patients.

### Active immune cells and immune checkpoint genes functional enrichment analysis

**Fig. 8:**
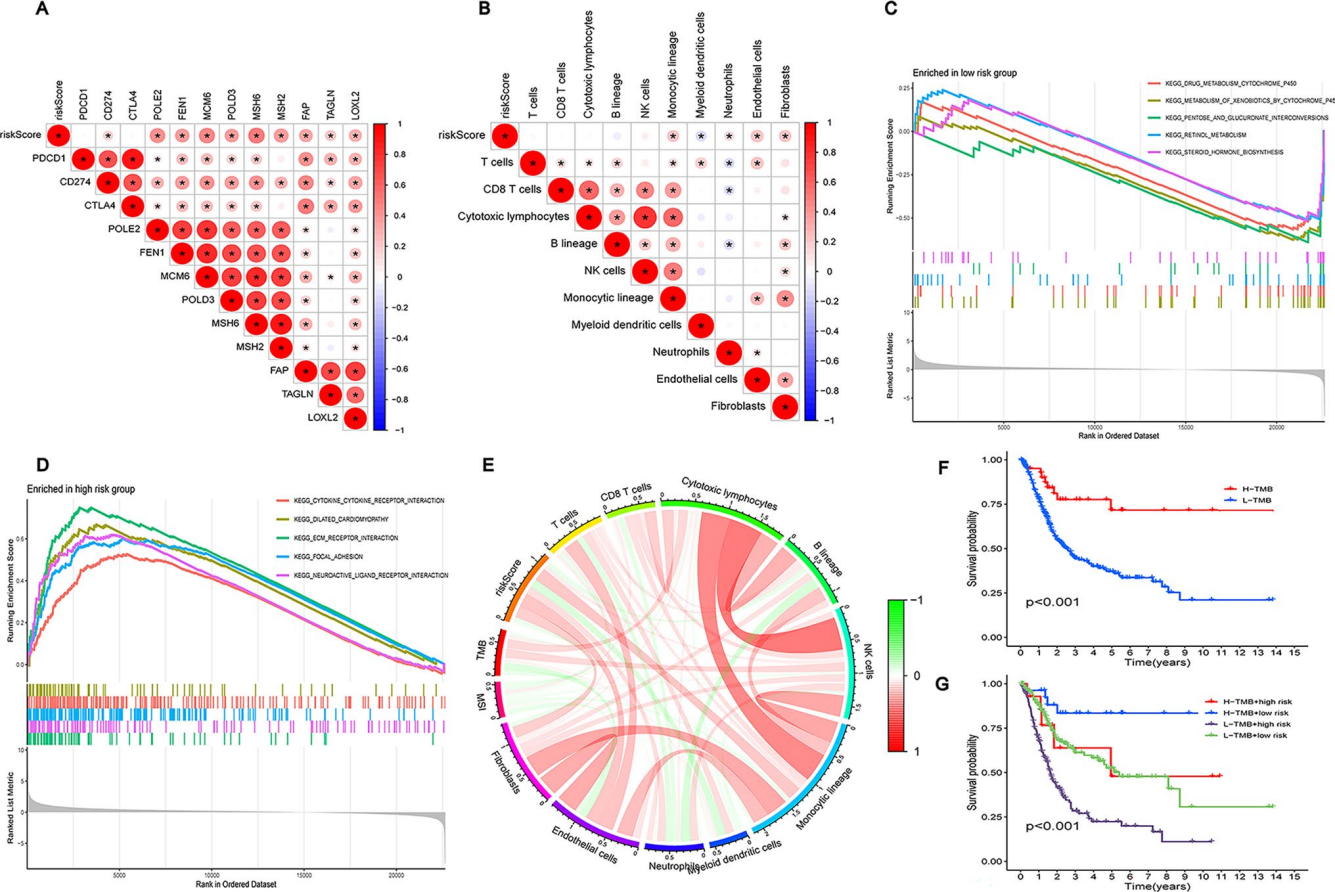
Active immune cells and immune checkpoint genes functional enrichment analysis. **A** Correlation heat map of risk score and immune checkpoint genes. **B** Correlation heat map of risk score and 10 types immune cell infiltrations. **C**–**D** Gene Set Enrichment Analysis (GSEA). **E** Tumor mutational burden correlates. **F**, **G** Survival analysis of tumor mutational burden

The correlation heatmap shows how the study examined the associations between risk scores and the 10 immune checkpoint genes levels of expression in bladder cancer (Fig. 8A). The correlation between risk scores and the expression of 12 important immune checkpoint genes was explored by further study. Surprisingly FEN1, LOXL2, MCM6, MSH2, MSH6, PDCD1, POLD3, POLE2, and TAGLN showed greater expression levels in the high-risk group and showed a positive connection with risk scores (P < 0.05) (Supplement Fig. 1).

Additionally, a correlation heatmap was used in the study to analyze the association between risk scores and the infiltration of 10 different immunocyte types (Fig. 8B**, **Supplement Fig. 2). According to the findings, the high-risk group of bladder cancer patients showed a much higher concentration of endothelial cells, cytotoxic lymphocytes, fibroblasts, monocytes, and NK cells. This reveals a close relationship between immunocyte levels and risk scores in the high-risk group, demonstrating the potential role of these immunocytes in modifying immune responses and affecting the onset and prognosis of BLCA. Endothelial cells contribute to angiogenesis and immune response regulation, while cytotoxic lymphocytes and NK cells are key immune cells involved in directly attacking and eliminating cancer cells. Fibroblasts and monocytes play roles in inflammation and immune regulation. Therefore, the elevated presence of these immunocytes in the high-risk group may reflect enhanced immunological responses against bladder cancer. Still, it could also signify more aggressive tumor behavior and poorer prognosis.

GSEA was used to find enriched pathways in each group to explore the molecular mechanisms underlying the prognostic differences between both groups. The top 5 enriched pathways (Table 1, Fig. 8C, D).

**Table 1:** The top 5 enriched pathways

The low-risk group	The high-risk group
STEROID_HORMONE_BIOSYNTHESIS	NEUROACTIVE_LIGAND_RECEPTOR_INTERACTION
RETINOL_METABOLISM	FOCAL_ADHESION
PENTOSE_AND_GLUCURONATE_INTERCONVERSIONS	ECM_RECEPTOR_INTERACTION
METABOLISM_OF_XENOBIOTICS_BY_CYTOCHROME_P45	DILATED_CARDIOMYOPATHY
DRUG_METABOLISM_CYTOCHROME_P450	CYTOKINE_CYTOKINE_RECEPTOR_INTERACTION

A circular plot (Fig. 8E) depicted the relationship between TMB, MSI, immunocytes, and risk scores. According to survival analysis, TMB and patient prognosis were correlated (Fig. 8F), with a higher TMB indicating a better prognosis. This relationship held for both groups (Fig. 8G).

### Better predictive performance of signatures from IMRGs than those from other studies

We conducted a comparative analysis of signatures developed by studies conducted by Wang, Hu, Lou, Peng, and Zou. The IMRG signature demonstrated a C-index of 0.667, whereas the Wang signature had a C-index of 0.611, the Hu signature had a C-index of 0.617, the Lou signature had a C-index of 0.579, the Peng signature had a C-index of 0.63, and the Zou signature had a C-index of 0.655 (Fig. 10A). These findings show that the IMRG signature outperforms the other four signatures regarding predictive performance.

**Fig. 9:**
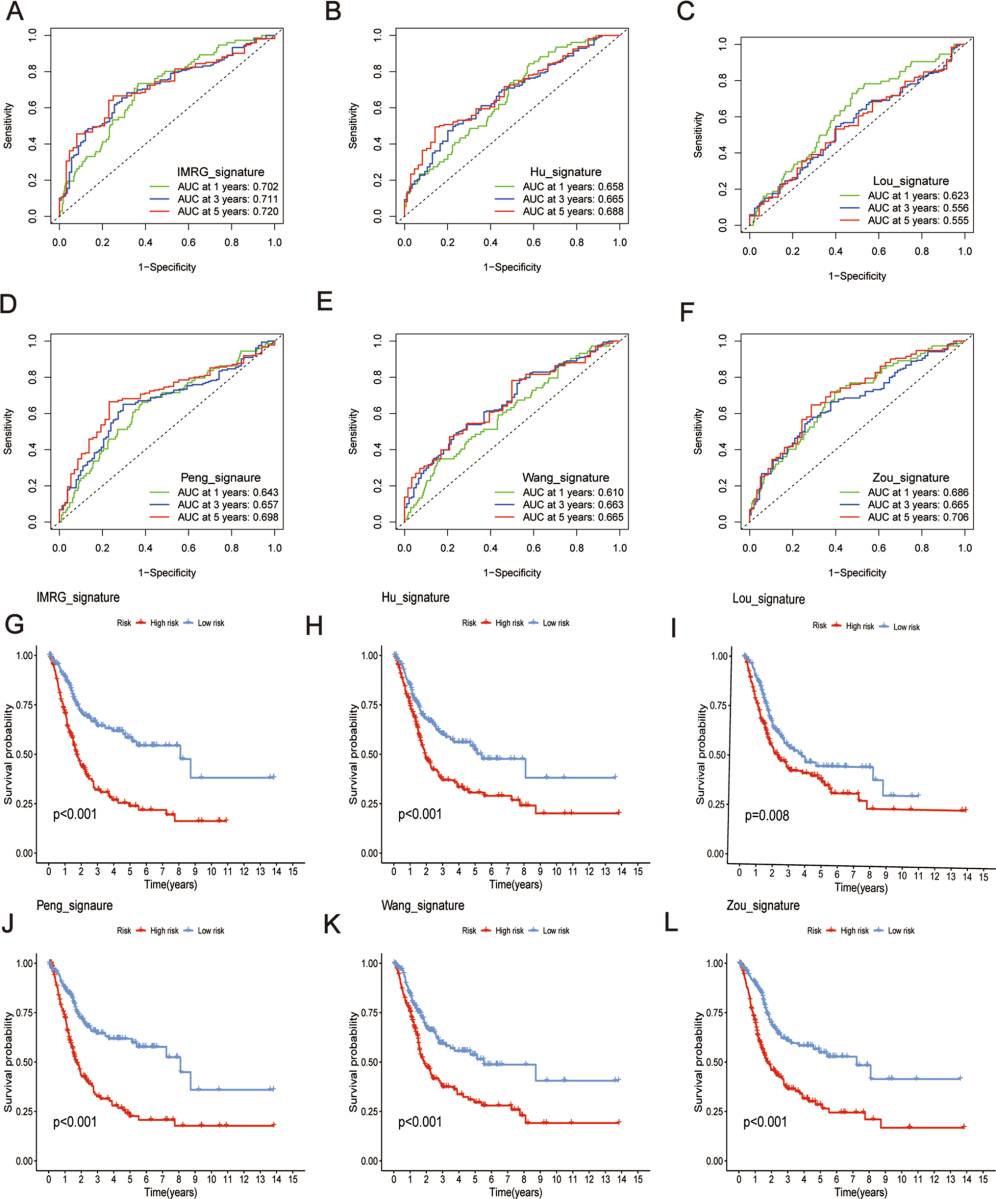
Better predictive performance of signatures from IMRGs than those from other studies. **A**–**F** AUC values of 1-year, 3-year, and 5-year ROC curves were calculated using IMRG and different research signatures. **G**–**L** KM curve of OS in IMRG signatures with varying signatures of research

Additionally, the analysis of the ROC curves for the IMRG signature demonstrated enhanced predictive accuracy relative to the other signatures, with AUC values of 0.702, 0.711, and 0.720 at 1, 3, and 5 years, respectively. The results presented here highlight the IMRG signature’s superior predictive capability compared to the other signatures (Fig. 9A–F). Moreover, we observed substantial disparities in overall survival among the risk groups defined by the IMRG, Wang, Hu, Lou, Peng, and Zou signatures (P < 0.005, Fig. 9G–L).

### Differences in immunotherapy in both groups

**Fig. 10:**
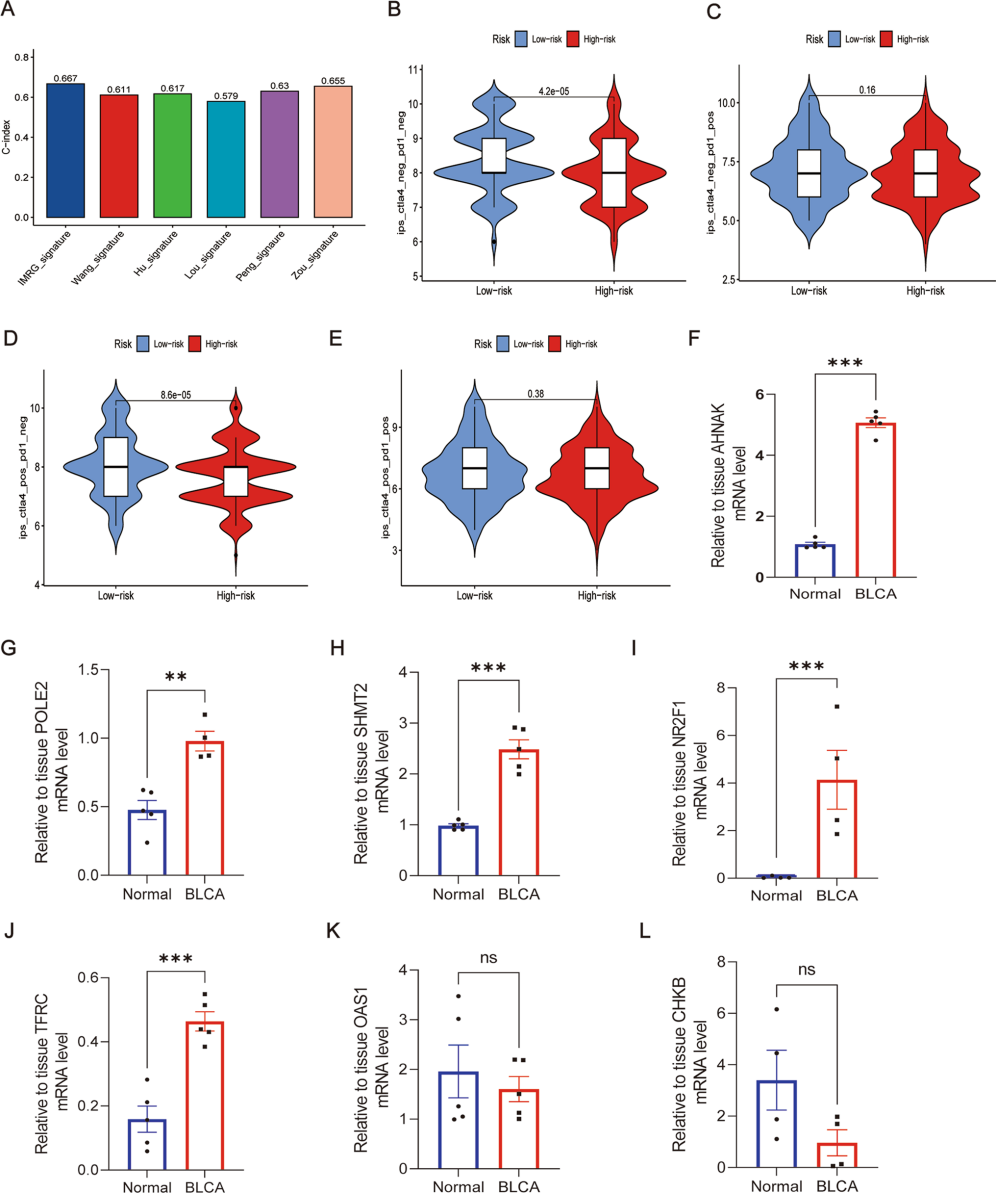
Immunotherapy response and verification of the prognostic gene expression by qRT-PCR. **A** Model comparison (C-index). **B**–**E** Analysis of Immunotherapy. **F**–**L** AHNAK, POLE2, SHMT2, NR2F1, TFRC, OAS1, CHKB qRT-PCR of bladder tissue, n = 4–5/group, *P < 0.05; **P < 0.01; ***P < 0.001

The analysis of immune therapy revealed that the expression of CTLA-4PD1^+^ and CTLA^+^4PD1^+^ did not show significant associations, while the CTLA^+^4PD1^−^ and CTLA^−^4PD1^−^ groups displayed notable differences in the efficacy of immune therapy (Fig. 10B–E). An important result was that the low-risk group responded better than the high-risk group, suggesting that patients in the low-risk group may experience greater benefits from immunological therapy.

### Verification of the prognostic gene expression by qRT-PCR and western blotting

The differential expression of prognostic genes was verified by applying qRT-PCR and western blotting to assess mRNA and protein expression in vivo. The findings showed that AHNAK, POLE2, SHMT2, NR2F1, and TFRC expression in tumor tissues was substantially higher than in normal tissues (P < 0.05, Fig. 10F–J, Supplement Fig. 3A, B). OAS1 and CHKB, on the other hand, showed a downregulation trend in their mRNA and protein expression, while the alterations were not statistically significant (P > 0.05, Fig. 10K–L, Supplement Fig. 3A, B).

## Discussion

In this investigation, we analyzed the expression patterns of immune and metabolism-related genes (IMRGs) in BLCA and examined their potential prognostic implications. Our outcomes align with previous research, as other researchers have also reported the presence of differentially expressed genes in BLCA [43]. For example, Zhang et al. conducted a study that identified a considerable number of genes exhibiting differential expression in bladder cancer, with some of them being closely linked to immune and metabolic processes [44–46]. These consistent outcomes support the exhaustive analysis of IMRGs conducted in our study.

We identified 49 IMRGs substantially associated with prognosis via univariate Cox regression analysis. Using clustering analysis, we categorized BLCA samples into two subgroups (C1 and C2) using these prognostic-associated genes. Following survival analysis revealed substantial differences between these subgroups regarding OS and PFS. Further examination of the TME confirmed that the C1 subgroup had increased immunocyte infiltration scores. The promise of immunotherapy as a fundamental strategy in the clinical treatment of many cancers is suggested by the critical function that immune cells play in eradicating cancer cells [12, 47].

To establish a robust prognostic signature, we conducted MCRanalysis and identified a prognostic signature comprising seven genes: POLE2, AHNAK, SHMT2, NR2F1, TFRC, OAS1, and CHKB. The POLE2 gene encodes the DNA polymerase epsilon subunit 2, vital in DNA replication and repair processes. POLE2 contributes to the synthesis of new DNA strands, ensuring the stability and integrity of the genome [48]. The disruption of DNA replication and repair caused by dysregulated POLE2 expression may have detrimental implications on the stability of the genome and cellular function [49, 50]. Abdel-Rahman N Zekri et al. initially found POLE 2 in bladder cancer, however the precise pathophysiology was not investigated [51]. However, it has been observed that POLE 2 knockdown impeded the growth of A549 and death of NCI-H1299 cells, which was also linked to treatment sensitivity in lung adenocarcinoma, rectal cancer, and esophageal cancers [48, 52–54].Thus, we speculate that the POLE 2 gene can both serve as a potential target gene for treatment and further increase the occurrence and development of the urinary bladder.AHNAK has shown altered expression levels. Numerous studies have reported a significant upregulation of AHNAK expression in bladder cancer tissues [55, 56]. The specific functional and molecular roles of AHNAK in bladder cancer are still unknown. Several studies, however, have provided insights into the cellular processes and pathways in which AHNAK may be involved. AHNAK is involved in cell adhesion, migration, and metastasis in bladder cancer [57, 58]. In addition, AHNAK may interact with the tumor microenvironment, invasion, and transformation of tumor cells [59–61]. Xie et al. discovered that cisplatin resistance generated by N-acetyltransferase 10 required AHNAK-mediated DNA damage repair [32]. Thus, cisplatin sensitivity may increase by targeting the AHNAK gene. Research on SHMT2 in BLCA has revealed its potential association. A study reported a notable increase in the expression level of SHMT2 in BLCA patients, indicating its possible involvement in regulating cell proliferation, cell cycle, and apoptosis in BLCA [62].

NR2F1, a gene encoding a transcription factor, is Nuclear Receptor Subfamily 2 Group F Member 1 or COUP-TFI (Chicken Ovalbumin Upstream Promoter Transcription Factor I). In the context of BLCA, dysregulated expression of NR2F1 has been verified in cancer cell proliferation, invasion, and metastasis [63]. The current research has demonstrated that agonists of NR2F1 can induce cancer cell dormancy to inhibit tumor metastasis[64]. In addition, for thyroid cancer, breast cancer, and others, NR2F1 is considered an oncogenic gene. However, the specific mechanism of NR2F1 in the occurrence of BLCA remains unclear. TFRC (Transferrin Receptor Protein 1), also called CD71, is a gene that encodes the transferrin receptor protein. Predictive models have indicated significant upregulation of TFRC expression in BLCA tissues, which correlates with clinical features and prognosis of the tumor [65, 66]. Su et al. have presented that circular RNA-cTFRC upregulated and correlated with tumor grading and low survival rates in BLCA patients [67]. We speculate that knocking down or interfering with TFRC may inhibit the occurrence and development of BLCA, thereby improving patient prognosis and survival rates. The overexpression of TFRC has been related to promoting BLCA invasion cell proliferation, invasion, and metastasis. The level of OAS1 gene expression is closely linked to tumor onset and progression [68]. According to research, OAS1 is significantly downregulated in BLCA tissues, and this downregulation is linked to the clinical characteristics and prognosis of the tumor [69, 70]. OAS1 enhances the immune cell’s capacity to fight viral infections by triggering the interferon signaling pathway. This immune reaction slows the growth of tumors and makes it easier to get rid of infected viruses. Studies have shown a strong association between the emergence and development of tumors and the CHKB gene's level of expression. Experimental research has shown that the expression of the CHKB gene is significantly reduced in BLCA tissues. CHKB plays essential roles in cellular processes such as choline metabolism, phosphatidylcholine biosynthesis, and phosphoethanolamine biosynthesis, which are closely associated with cell growth, differentiation, and metabolic regulation [71, 72]. Consequently, the aberrant expression of CHKB may influence the growth and metabolism of tumor cells. Currently, no study has reported the mechanistic relationship between CHKB and BLCA. We suppose that its expression may influence the growth and metabolism of BLCA, thus further affecting the occurrence and development of BLCA. Taken together, above genes impact the growth and metabolism of BLCA to a certain extent, suggesting them as potential therapeutic targets for BLCA.

Additionally, our data showed a strong correlation between increasing risk scores and older age, male gender, higher grade, and advanced stage, indicating a poor prognosis. These findings highlight the significance of considering these characteristics when making clinical decisions and creating individualized treatment plans. Additionally, our study showed relationships between risk scores and enriched pathways, immunocyte infiltration, and immunological checkpoint genes. Moreover, the abundance of specific immunocyte types was related to risk scores, suggesting their involvement in modulating immune responses and influencing the development and prognosis of BLCA. GSEA also revealed probable biological pathways that might be causing prognostic variations between both groups. The enriched pathways in the low-risk group primarily involve drug metabolism, xenobiotic metabolism, and hormone biosynthesis, while the high-risk group presented enrichment in pathways associated with cytokine signaling, cardiac function, and extracellular matrix interactions. Dihydrotestosterone (DHT), a type of steroid hormone, has been demonstrated by Yang et al. to promote proliferation and invasion of bladder cancer cells through the MAPK/JUP signaling pathway [73]. Previous studies have found an association between retinoic acid and bladder cancer [74]. A research has revealed that the expression of retinol acyltransferase and the high methylation of CpG in retinol-binding protein 1 contribute to the further development of bladder cancer [75, 76]. These studies also demonstrate significant therapeutic potential of retinoic acid in the prevention and treatment of BLCA. Currently, there is no further explorations about the relationship between pentose and glucuronate interconversions and the occurrence and development of BLCA. By urinary metabolomics in urothelial carcinoma, Yang et al. revealed the dysregulation in pentose and glucuronate interconversions, which was significantly associated with the survival rate of bladder cancer [77]. Cytochrome P450 is involved in the metabolism of various carcinogens. Relevant studies have confirmed that inhibiting cytochrome P450 can exhibit anti-proliferative and apoptotic effects on bladder cancer cells [78]. The interactions between cytochrome P450 and patient serum could predict bladder cancer [79]. In the pathways enriched in the high-risk group, especially the pathways related to the interaction of neuroactive ligand-receptor and cytokine-cytokine receptor, were found significant enriched by Zhang. The genes within these pathways exhibit significant diversity in bladder cancer subtypes [80]. Adherens junction kinase is protein tyrosine kinase located in adherens junctions. It plays a crucial role in the adhesion and invasion of bladder cancer cells. It can regulate the transforming growth factor-beta (TGFβ) and modulate the phosphoinositide 3-kinase (PI3K)/Akt signaling pathway, thereby mediating cell migration and apoptosis [81, 82]. Therefore, this kinase can serve as a novel target for treating bladder cancer. The extracellular matrix (ECM) is involved in numerous biological functions about tumor initiation and development. Activating ECM can enhance bladder cancer cell proliferation and angiogenesis, thereby increasing the invasiveness of tumor cells’ [83].These results emphasize the important roles these pathways play in the development and prognosis of BLCA.

In order to predict the survival rate of BLCA patients, we developed a prognostic signature in our study based on the expression of immune and metabolism-related genes (IMRGs). However, our findings are subject to certain limitations. Firstly, our study's GEO and TCGA datasets exhibit inherent heterogeneity and potential selection bias, which may introduce some degree of bias into the results. Secondly, we solely validated our findings using RT-PCR testing on tissue samples without further investigating the underlying mechanisms of key genes or immune-related factors in BLCA through in vitro or in vivo experiments. Further clinical research is necessary to confirm these results and evaluate the potential use of this predictive characteristic in guiding immune therapy for BLCA patients, even though our investigation shed light on the responsiveness to immune therapy. Therefore, additional research is warranted to address these limitations and further enhance our understanding of the prognostic implications and therapeutic potential of IMRGs in BLCA.

## Conclusions

The first predictive signature based on immune and metabolism-related genes (IMRGs) that divides BLCA patients into two different subgroups with statistically significant variations in survival outcomes has been created and confirmed by our team. The established model serves as a novel assessment tool for predicting prognosis and evaluating the efficacy of immune therapy in BLCA patients. These findings hold promise for gaining insights into developing novel immune biomarkers and targeted therapies in BLCA.

### Supplementary Information

Additional file1 (TIF 23514 KB)

Additional file 2 (TIF 23667 KB)

Additional file3 (DOCX 312 KB)

Additional file4 (DOCX 16 KB)

## Data Availability

The datasets generated during and/or analysed during the current study are available from the corresponding author on reasonable request.
