# Supplementary figures and images for: BLCA prognostic model creation and validation based on immune gene-metabolic gene combination
Source: Discov Oncol. 2023 Dec 16;14:232. doi: 10.1007/s12672-023-00853-6 (PMC10725402; doi:10.1007/s12672-023-00853-6)

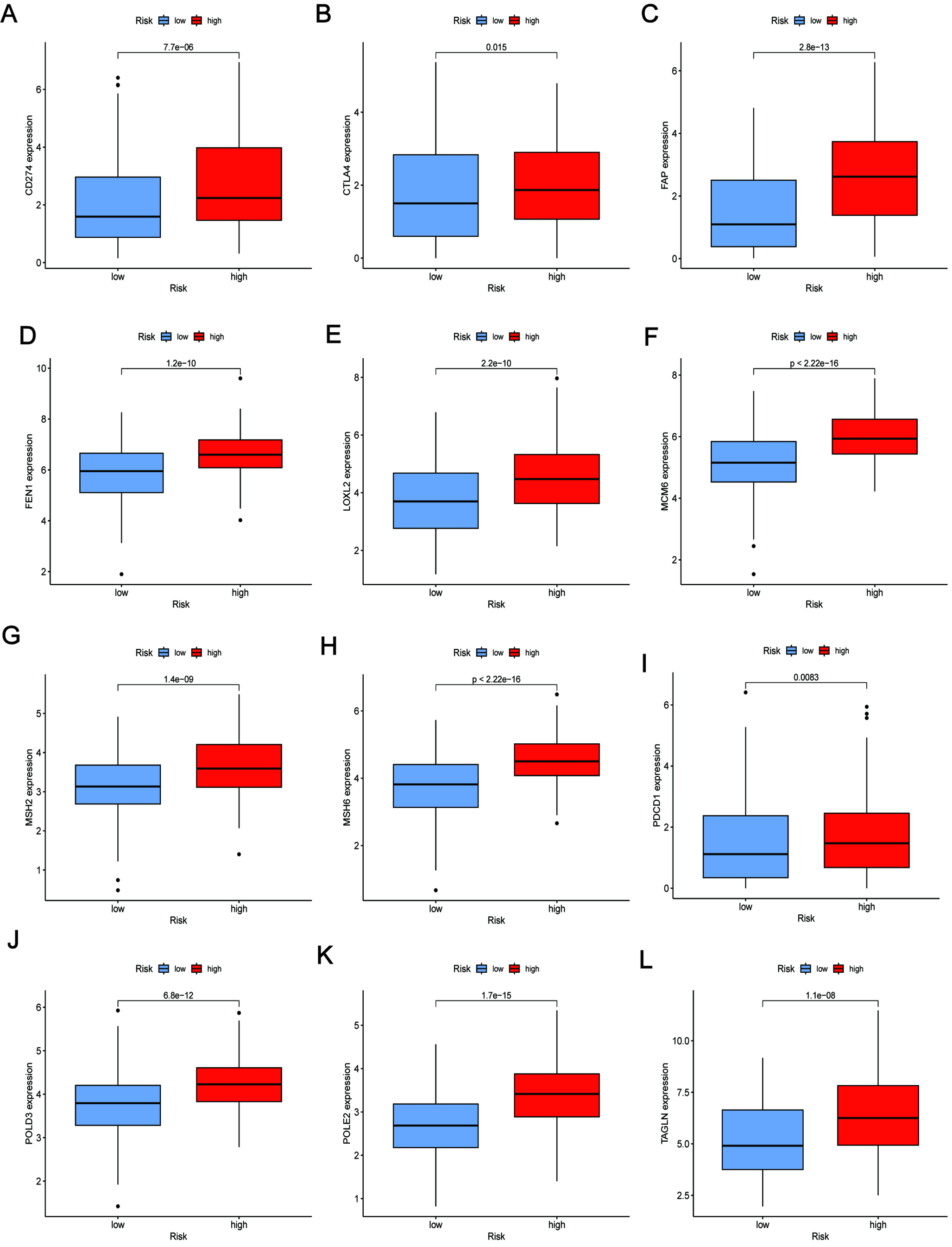

Supplement: Supplementary file 1 — Additional file1 (TIF 23514 KB) [file 12672_2023_853_MOESM1_ESM.tif]

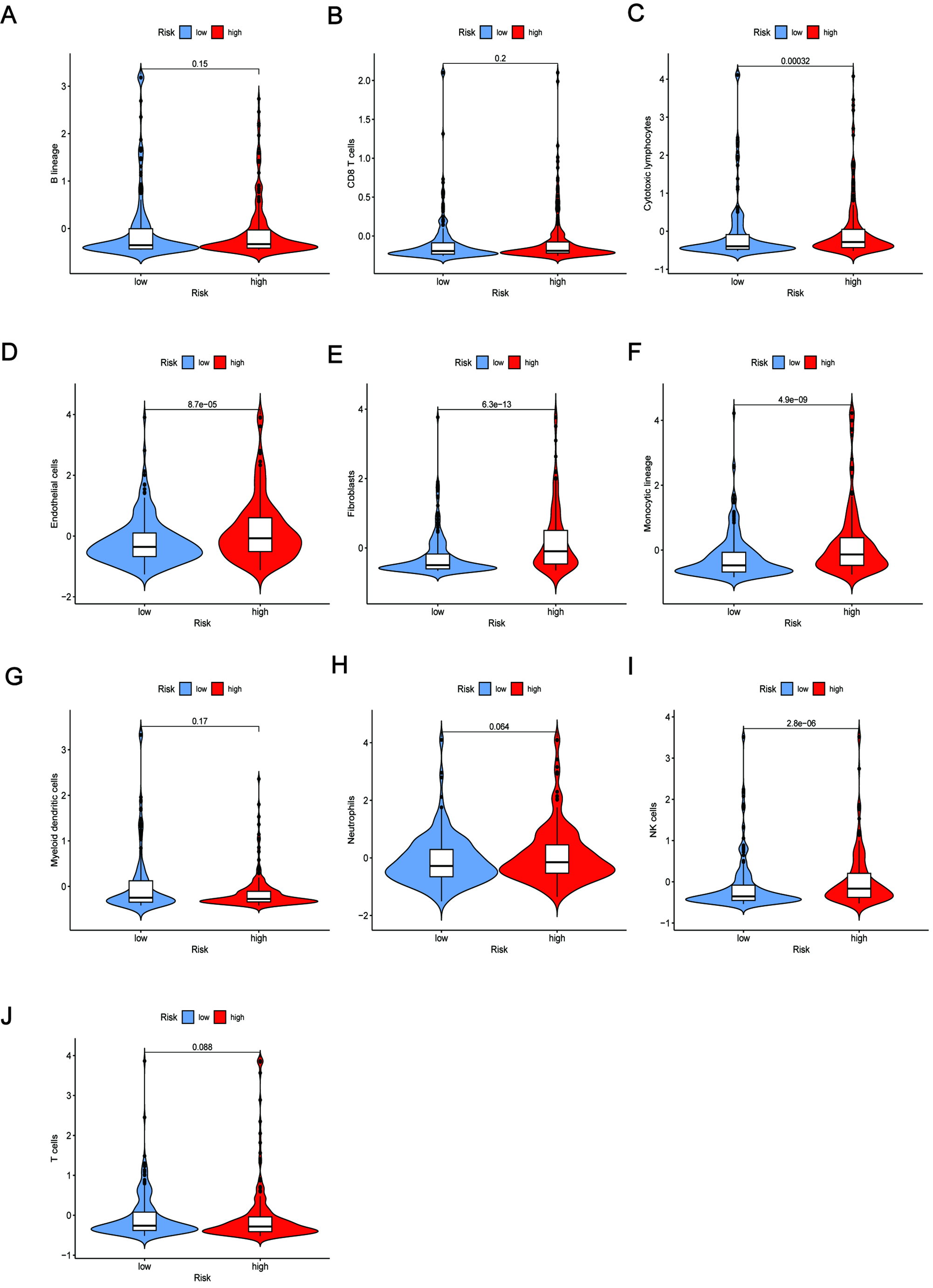

Supplement: Supplementary file 2 — Additional file 2 (TIF 23667 KB) [file 12672_2023_853_MOESM2_ESM.tif]
