# Supplementary material for: BLCA prognostic model creation and validation based on immune gene-metabolic gene combination
Source: Discov Oncol. 2023 Dec 16;14:232. doi: 10.1007/s12672-023-00853-6 (PMC10725402; doi:10.1007/s12672-023-00853-6)
Supplement: Supplementary file 3 — Additional file3 (DOCX 312 KB) [file 12672_2023_853_MOESM3_ESM.docx]

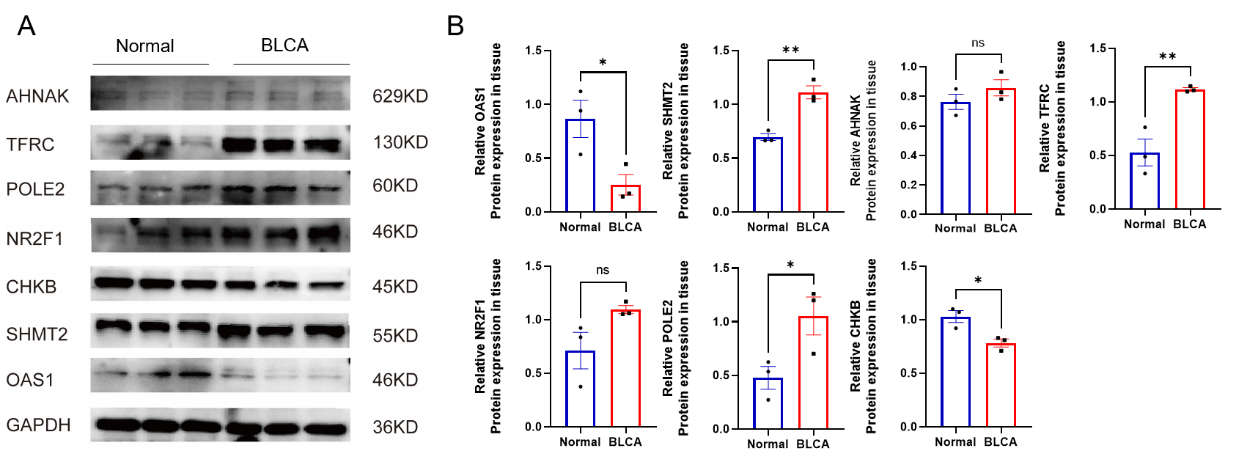
Supplement Figure 3: Verification of the prognostic gene expression by western blotting(A-B) AHNAK, POLE2, SHMT2, NR2F1, TFRC, OAS1, CHKB western blotting of bladder tissue, n=3/group, *, P<0.05; **, P<0.01; ***, P<0.001.
