# Supplementary material for: BLCA prognostic model creation and validation based on immune gene-metabolic gene combination
Source: Discov Oncol. 2023 Dec 16;14:232. doi: 10.1007/s12672-023-00853-6 (PMC10725402; doi:10.1007/s12672-023-00853-6)
Supplement: Supplementary file 4 — Additional file4 (DOCX 16 KB) [file 12672_2023_853_MOESM4_ESM.docx]

**Supplement table Human primers for quantitative Real-Time PCR.**

| Gene |  | Sequence 5’----3’ |
| --- | --- | --- |
| POLE2 | Foward | TGAGAAGCAACCCTTGTCATC |
|  | Reverse | TCATCAACAGACTGACTGCATTC |
| AHNAK | Foward | TACCCTTCCTAAGGCTGACATT |
|  | Reverse | TTGGACCCTTGAGTTTTGCAT |
| SHMT2 | Foward | CCCTTCTGCAACCTCACGAC |
|  | Reverse | TGAGCTTATAGGGCATAGACTCG |
| NR2F1 | Foward | ATCGTGCTGTTCACGTCAGAC |
|  | Reverse | TGGCTCCTCACGTACTCCTC |
| TFRC | Foward | ACCATTGTCATATACCCGGTTCA |
|  | Reverse | CAATAGCCCAAGTAGCCAATCAT |
| OAS1 | Foward | TGTCCAAGGTGGTAAAGGGTG |
|  | Reverse | CCGGCGATTTAACTGATCCTG |
| CHKB | Foward | GGCTTGCAGCAGTCTAAGTG |
| GAPDH | Reverse  Foward  Reverse | GGCACCATTGGTAGGCTCG  GGAGCGAGATCCCTCCAAAAT  GGCTGTTGTCATACTTCTCATGG |
